# C-reactive protein point-of-care testing and antibiotic prescribing for acute respiratory tract infections in rural primary health centres of North Ethiopia: a cross-sectional study

**DOI:** 10.1038/npjpcrm.2015.76

**Published:** 2016-01-14

**Authors:** Henock Yebyo, Araya Abrha Medhanyie, Mark Spigt, Rogier Hopstaken

**Affiliations:** 1 School of Public Health, College of Health Sciences, Mekelle University, Mekelle, Ethiopia; 2 Department of Family Medicine, School for Public Health and Primary Care (CAPHRI), Maastricht University, Maastricht, The Netherlands; 3 General Practice Research Unit, Department of Community Medicine, the Arctic University of Norway, Tromsø, Norway; 4 Saltro Diagnostic Centre, Utrecht, The Netherlands

## Abstract

Unjustified antibiotic prescribing for acute upper respiratory infections (URTIs) is probably more common in poor-resource settings where physicians are scarce. Introducing C-reactive protein (CRP) point-of-care testing in such settings could reduce the misuse of antibiotics, which could avert antibiotic resistance. However, information useful for the applicability of CRP test in resource-limited settings is lacking. This study aimed to elicit the frequency of antibiotic prescribing and distribution of CRP levels in remote, rural settings in Ethiopia. We included 414 patients with acute URTIs from four health centres. Health professionals recorded the clinical features of the patients, but the laboratory professionals measured the CRP levels of all patients at the point of care. The most prominent respiratory causes for consultation were acute URTIs combined (44.4%), and lower respiratory tract infections—pneumonia (29.71%) and acute bronchitis (25.84%). The CRP distribution was <20 mg/l, 20–99 mg/l and 100 mg/l or more in 66.6%, 27.9% and 5.5% of the patients, respectively. The CRP levels were significantly different among these clinical diagnoses (*X*^2^=114.3, *P*<0.001, d.f.=4). A wide range of antibiotics was administered for 87.8% of the patients, regardless of the diagnostic or prognostic nature of their diseases. Antibiotic prescribing for acute URTIs in the rural areas of Ethiopia is unduly high, with high proportions of mild, self-limiting illness, mostly URTIs. Implementation of CRP point-of-care testing in such resource-constrained settings, with low- or middle-grade healthcare professionals, could help reconcile the inappropriate use of antibiotics by withholding from patients who do not benefit from antibiotic treatment.

## Introduction

Acute respiratory tract infections (RTIs), both upper (URTI) and lower, are among the most common reasons to consult in primary care and a frequent reason to prescribe antibiotics. Most of these prescriptions are not helpful and only contribute to the emerging problem of bacterial resistance.^1–3^

The use of tonnes of antibiotics over the past years has made almost all disease-causing bacteria resistant to antibiotics commonly used to treat them.^4,5^ Studies show that improper use of antibiotics is more pronounced in acute respiratory infections to which antibiotics do not meaningfully change important outcomes.^6,7^ Diagnostic (viral or bacterial cause) or prognostic (life-threatening or self-limiting infection) uncertainty makes it difficult for clinicians to know when to prescribe and when to withhold antibiotic treatment.^5,6^ Consequently, antibiotics are overused in hospitals and outpatient settings, resulting in increased antibiotic resistance^8^ and the pandemic spread of highly resistant bacterial clones.^9^ The effects of antibiotic resistance on human health are probably highest in countries with the lowest income, because the spread of resistant bacteria is facilitated by poor hygiene, contaminated food, polluted water, overcrowding and increased susceptibility to infection resulting from malnutrition or HIV.^3^

Acute respiratory tract infections are the leading reason for outpatient visits and prescription of antibiotics for both adults and children in primary healthcare units (PHUs) in Ethiopia.^10^ Health workers in primary healthcare settings in Ethiopia are not medical doctors, but usually nurses, midwives or health officers.^11^ Additional investigations like laboratory testing and chest radiography are neither accessible nor feasible.^12^ The urge for simple, cheap and accurate tools that could help to create better antibiotic stewardship is evident.^1,13^

C-reactive protein (CRP), an acute-phase reactant, is a diagnostic marker that helps to differentiate serious infections like pneumonia and sepsis from self-limiting illnesses.^14–16^ The effect of CRP point-of-care testing (POCT) in reducing antibiotic prescribing for lower respiratory tract infections has been demonstrated in developed countries.^1,2,17^ Despite its potential, however, CRP POCT is not widely used in resource-limited countries.^17^ The nature and severity of diseases presented in primary care in low-income countries could be very different from developed countries. If, for example, people have to travel a long way to a health centre, they may only visit the health centre with very severe diseases. This could make the use of CRP testing less relevant.^6^

Therefore, this study aimed to study the distribution of CRP levels for acute URTIs and the frequency of antibiotic prescribing to patients presented with these diseases in Primary Health Care Units in rural Northern Ethiopia. The findings could help to decide whether the CRP POCT could assist health professionals in diagnosis and antibiotic prescribing during consultation in low-income countries.

## Results

### Socio-demographic and medical description of the study patients

We included 414 adult patients with acute URTIs. The average age of the patients was 40.3 years. The majority of them were illiterate (239, 59.6%). Almost equivalent numbers of patients were recruited from the four health centres (Table 1). Twelve health professionals participated: six bachelor-degree-holding nurses, four midwives and two health officers.

### Medical presentations

All patients had at least one of the symptoms of acute URTIs. With regard to the compulsory inclusion criteria, the most frequent medical symptom was acute cough, which was presented in 83.2% of the patients and accounted for nearly one-third of all the signs/symptoms (34.4%), whereas 8.4% of them had worsening chronic cough. The rest, 8.4%, did not have clear symptoms in terms of time of having cough, acute versus chronic.

The patients had at least one of the four major clinical features—shortness of breath, wheezing, chest pain and auscultation abnormalities. Out of the generalised symptoms, the most frequent were headache and chest pain, which presented in 134 (46.4%) and 138 (33.2%) patients, respectively (Table 2).

### Diagnoses and CRP distribution

The top clinical diagnoses recorded by the healthcare professionals, independent of the CRP levels, were acute URTIs combined (44.4%), followed by pneumonia (29.6%) and acute bronchitis (25.8%).

**Figure 1:**
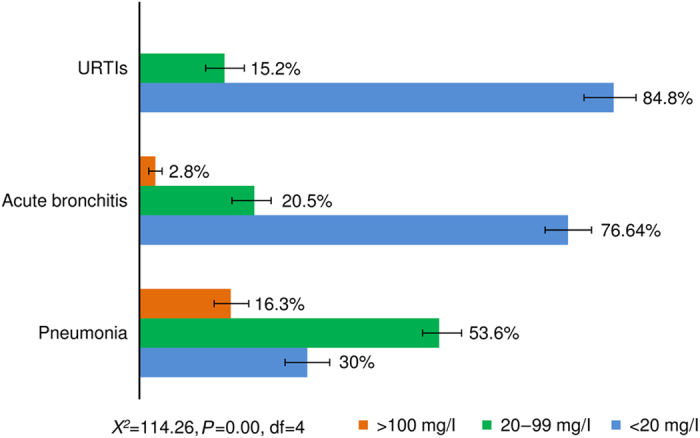
The respiratory diseases by CRP distributions and 95% confidence interval of the patients in four health centres, Tigray, North of Ethiopia.

Overall, the median CRP level was 11 mg/l (interquartile range: 8–29). An overall 66.6% of patients had test results less than 20 mg/l; 27.9% had between 20 and 99 mg/l, and 5.5% had 100 mg/l or more (Figure 1). The distribution of CRP over the different diagnoses showed that the majority of patients had low CRP levels. In particular, a low CRP value was observed among patients with unspecified acute URTIs (Figure 1). Statistically, there was a significant difference in the CRP measurements among the major diagnoses—acute bronchitis, pneumonia and unspecified acute URTIs (*X* ^2^=114.3, *P*<0.001, d.f.=4; Figure 1).

Along with the current medical presentations, 16% of the patients reported at least one other (chronic) illness. The most frequently reported illness was chronic bronchitis, in 25 (6.0%) patients, followed by peptic ulcer disease (PUD), reported by 8 (1.9%) patients (Table 3).

### Antibiotic prescribing

The health providers prescribed a wide range of antibiotics in 87.8% of the cases and other drugs such as vitamin C and analgesics in 7.4% of the patients. They provided reassurance only to 4.8% of the patients. Amoxicillin was prescribed in 52.16% of patients, Doxycycline in 11.3%, Erythromycin in 6.2%, analgesics in 5.5%, Ciprofloxacin and Contrimoxazole each in 3.8%, and others in 17.2% (Table 4). In addition, 232 (55.7%) patients were administered additional drugs. The most frequently prescribed drugs as an addition to the primary medications were analgesics (69.8%), Diphenhydramine (5.3%), Dexamethasone and Neomycin (4.80%) and Vitamin B complex (3.06%).

## Discussion

### Main findings

On the basis of the usual care—i.e., without the use of CRP levels for management decisions – the study found that irrational antibiotic prescribing for acute URTs was very high in Ethiopia. Almost all patients received antibiotics, despite low severity and low CRP values. The CRP distribution was similar to what has been observed in European primary care studies on lower respiratory tract infections.

### Strengths and limitations of this study

#### Strengths

Irrational use of antibiotics is assumed to be high in low-resource countries, which demands immediate remedy to halt expansion of antibiotic-resistant microbes. As such, we believe that the study is the first of its kind in low-resource countries to test the feasibility of CRT POCT. This would have high relevance for implementation of the POCT in resource-limited settings to reduce unnecessary antibiotic prescribing. In addition, the study focussed on the high World Health Organization (WHO) priority to address the problem of bacterial resistance and to find ways to compact it. The study also followed a pragmatic approach that it did not violate the routine services for the patients. The health professionals were blind to the cutoff values of CRP POCT (as set in other countries), and the test was done after health professionals had finished their contacts with the patients to avoid unbiased management.

#### Limitations

The study may have limitations in that it is basically a feasibility study and it would not prove that CRP POCT helps for better antibiotic stewardship. It needs a randomised controlled study to test whether CRP POCT can safely reduce antibiotic prescriptions for acute URTIs in order to contribute to the fight against antibacterial resistance. Selection bias of recruited patients cannot be excluded; that is, we included all patients who fulfilled the inclusion criteria and were presented to the study health centres during the study period. However, we believe that the presence of the patients to the health facility is independent of the types and severity of the presented illness; thus, this might not violate the estimated CRP distribution and antibiotic prescribing.

### Interpretation of findings in relation to previously published work

Although the patients from developing countries were hypothesised to have high levels of CRP because of the prevalence of infectious diseases, the current study found that the distribution for LRTs is comparable to that in patients in the Netherlands, where 69%, 24% and 7% of patients had <20 gm/l, 20–99 gm/l and >100 gm/l, respectively.^2^

The frequent causes of consultation in the study areas were acute URTIs followed by pneumonia and bronchitis. The majority of the patients were administered antibiotics regardless of the severity or aetiology of the diseases.

In addition to the high uncertainty nature of the clinical features to identify patients with viral or bacterial respiratory infections and poor or no investigative facilities, the limited training of health professionals in the PHU of Ethiopia could explain the misuse of antibiotics. As revealed in clinical trials in developed countries, only severe respiratory tract infections warrant antibiotic treatment.^1,2^ Clinical trials show that patients with lower CRP levels (<20 mg/l) were prescribed fewer antibiotics than those with higher CRP levels,^1,2,18,19^ assuring that all patients do not need antibiotics and the CRP cutoff points helped the health professionals to decrease the antibiotics prescribing. In the current study, the range of CRP levels of the patients were comparable to those in previous European studies, but antibiotic prescribing was much higher and generally regarded as inappropriately high. This indicates that unnecessary antibiotics prescribing could be safely reduced if CRP point-of-care testing is implemented.

With respect to diagnoses, the distribution of CRP levels in the current study was higher in the clinically diagnosed pneumonia cases. The distribution of CRP levels among the respiratory diagnoses is similar to studies conducted in developed countries, which reported that pneumonia cases had higher levels of CRP than the acute bronchitis cases and acute URTIs.^13,18^ Studies show that CRP cutoff points have importance in indicating pneumonia cases. This was found effective to decrease unnecessary antibiotic prescribing.^13^ CRP point-of-care testing would therefore be helpful in poor-resource countries for assisting in diagnosis and antibiotic prescribing; it would have paramount advantage in assisting especially middle-grade healthcare professionals.

### Implications for future research, policy and practice

The study results indicate the presence of huge antibiotic overprescribing for mostly mild illnesses. This implies a high potential use of CRP POCT in primary healthcare units of resource-limited settings to help direct antibiotic prescribing together with better education and training of health workers and the public. However, other factors including the health workers’ attitudes towards diagnostic testing, patients’ attitude towards antibiotics prescribing, approval by regulatory authorities, recommendation by guidelines, and social, ethical, economical and political factors could affect the uptake of the CRP point-of-care testing in such settings and thus should be studied before its implementation.

The high antibiotic prescribing is clearly a flaw that can contribute to antibiotic resistance. Furthermore, the effects of antibiotic resistance on human health are probably highest in countries with the lowest income, because the spread of resistant bacteria is facilitated by poor hygiene, contaminated food, polluted water, overcrowding and increased susceptibility resulting from malnutrition or Tuberculosis/Human Immunodeficiency Virus.^5^ As such, other studies are required to investigate the resistance level and sensitivity tests to the common antibiotics in use in Ethiopia.

Basically, the study is a feasibility study. This may not prove the effect of CRP POCT in helping health professionals prescribe appropriate antibiotics and reduce antibiotics. Yet, the results strongly suggest the need for randomised clinical trials on CRP POCT and training with antibiotic prescribing as a primary outcome measure.

### Conclusions

Antibiotic prescribing for acute URTIs in the Primary Health Care Units of Ethiopia is unduly high, with high proportions of mild, self-limiting illness, mostly URTIs. Implementation of CRP point-of-care testing in such resource-constrained settings with low educational level of health professionals could help reconcile the inappropriate use of antibiotics by withholding from patients who do not benefit from antibiotic treatment. In this way, Ethiopia could prevent the unnecessary use of scarce health resources and contribute to the fight against antibacterial resistance.

## Materials and methods

### Study settings

The study was undertaken in the Primary Health Care Units of Tigray region, Northern Ethiopia. Four rural health centres were included. In Ethiopia, a health centre is structured to offer curative outpatient treatment and preventive services to nearly 25,000 people. They are equipped with some basic laboratory facilities and basic pharmacies. Nurses, health officers and midwives are assigned to diagnose and treat patients. The health officer programme is a Bachelor's degree that trains students for 4 years on medical practice, including diagnosis, investigation, treatment and disease prevention.

### Study design

This is a cross-sectional registration study that describes clinical characteristics, routine management and distribution of CRP point-of-care test results of patients visiting primary healthcare centres with acute URTIs in rural Ethiopia.

### Study population and procedures

All adult patients who presented with clinical features of an acute respiratory infection were considered for the study. The inclusion criteria were categorised as compulsory, secondary and generalised clinical features. Patients aged 18 years and over with a new (i.e., <21 days) or worsening cough, combined with at least one of the following four secondary clinical features, namely, shortness of breath, wheezing, chest pain and auscultation abnormalities, and one of the generalised symptoms, namely, fever, perspiration, headache and myalgia, were eligible to enter the study. On the basis of the availability of CRP test kits from Saltro Diagnostic Centre, we included 414 patients in our study. We included an equal proportion of patients from each health centre.

The health workers responsible for diagnosing and treating patients in the health centres were trained to identify eligible patients for the test and request the CRP test. However, they were not given the CRP cutoff points for indications for treatment to avoid unbiased investigation of usual care (care that would have been given without the CRP test) and the test was conducted after contact with the patients.

CRP was measured by the NycoCard CRP test (http://www.alere.com/ww/en/product-details/nycocard-crp.html), a 2-min, quantitative immunometric test with a measuring range of 8–200 mg/l, run on the NycoCard Reader II instrument. Considering positive CRP concentrations to be >10 mg/l, the test has good specificity of 83.3% and sensitivity of 94.4% when compared with our CRP-Lab.^20^ The instrument is easy to use, portable, and operates on batteries or electricity. The test requires one drop of blood from the finger. The laboratory technicians in each of the health centres carried out the procedure after a laboratory expert trained them on performing the procedure and on test precautions including proper storage and errors in reading.

### Analyses

We analysed the data using Stata 11 (Stata Corporation, College Station, TX, USA). We explored the data for errors and assumption fulfilments. The proportions of the CRP levels, patterns of acute URTIs and antibiotic prescribing levels were analysed using descriptive statistics. We computed the CRP levels using the cutoff points for clinical relevance, set in clinical trials and used in the guidelines on acute cough of the Dutch College of General Practitioners, and the United Kingdom National Institute for Health and Care Excellence guideline Pneumonia: Diagnosis and management of community- and hospital-acquired pneumonia in adults.^21,22^ The cutoff points were <20 mg/l, 20–99 mg/l and >100 mg/l. The statistical significance of the difference of the CRP measurements among the diagnoses was tested using the *χ* ^2^-test at *P*-value less than 0.05.

### Ethical consideration

Ethical approval was obtained from the College of Health Sciences of Mekelle University, Ethiopia, with reference number ERC 034/2013. As the literacy rate is low in the study area, we relied on verbal consent. After explanation of the purpose, information required from them, and the procedure of the test, including the need of a drop of blood from their fingers, verbal consent was obtained from each patient. The patients were informed about their right to withdraw from the study at any stage and that no patient-identifying attributes would be encoded and reported in any communication of the findings to keep their data confidential.

## Figures and Tables

**Table 1:** Socio-demographic characteristics of the study patients

*Characteristics*	*Category*	*Frequency (%)*
Sex (*n*=411)^a^	Male	198 (48.2)
	Female	213 (51.8)
Marital status (*n*=391)^a^	Single	117 (29.9)
	Married/living with partner	248 (63.4)
	Divorced	15 (3.8)
	Widowed	11 (2.8)
Age (*n*=386)^a^	Average	40.3 years
Educational level (*n*=399)^a^	No education	238 (59.6)
	Primary (1–6)	94 (23.5)
	Junior (7–8)	15 (3.7)
	Secondary/high school (9–12)	42 (10.5)
	College or above	10 (2.5)
Health facility (*n*=414)	Negash Health centre	105 (25.2)
	Agulae Health centre	101 (24.5)
	Hiwane Health centre	109 (26.2)
	Adigudem Health centre	99 (24.0)

a There were missing values.

**Table 2:** The distribution of medical presentations among the patients

*Inclusion clinical features*	*Symptoms and signs*^a^	n	*% out of all recorded symptoms and signs*	*% of patients*
Compulsory	Acute cough	346	33.1	83.2
	Worsening chronic cough	35	3.3	8.4
	Acute cough/worsening chronic cough (unclear)	33	3.2	7.9
Secondary	Chest pain	138	13.2	33.2
	Difficulty of breathing	81	7.7	19.5
	Abnormal auscultation	36	3.4	8.6
	Wheezing	20	1.9	4.8
Generalised symptoms	Perspiration	22	2.1	5.3
	Headache	193	18.5	46.4
	Fever	134	12.8	32.2
	Myalgia	08	0.7	
	Total	1046	100	N/A

a A patient could have more than one medical presentation; thus, the total is not equal to the number of patients.

**Table 3:** The proportion of reported comorbidity among the patients

*Comorbidity*	*Frequency (%)*
Chronic bronchitis	25 (6.01)
Peptic Ulcer Disease	8 (1.92)
Arthritis	7 (1.68)
Hypertension	6 (1.44)
Osteomyelitis	6 (1.44)
Malaria	4 (0.96)
Bronchial asthma	3 (0.72)
Intestinal parasitosis	3 (0.72)
HIV	2 (0.48)
TB	2 (0.48)
Total	102 (24.5)

**Table 4:** The primary treatments prescribed for the patients

*Drugs*	*Frequency (%)*
Amoxicillin	217 (52.2)
Doxycycline	47 (11.3)
Erythromycin	26 (6.2)
Ampicillin	23 (5.5)
Analgesics (Diclofenac, Ibuprofen, ASA, Paracetamol)	23 (5.5)
Norfloxacin	19 (.2)
Ciprofloxacin	16 (3.8)
Cotrimoxazole	16 (3.8)
Cloxacillin	5 (1.2)
Chloramphenicol	4 (1.0)
Dexamethasone and Neomycin	4 (1.0)
Reassurance	20 (4.8)
Vitamin C	4 (0.9)
Others (Augmentin, Ceftriaxone, Dextromethadone, Gentamycin, Penicillin, Diphenhydramine)	10 (2.3)
Total	414 (100)

## References

[bib1] Cals, J. et al. C-reactive protein point-of-care testing for lower respiratory tract infections: a qualitative evaluation of experiences by GPs. Fam. Pract. 27, 212–218 (2010). 20022909 10.1093/fampra/cmp088

[bib2] Cals, J. , Butler, C. , Hopstaken, R. , Hood, K. & Dinant, G. -J. Effect of point of care testing for C reactive protein and training in communication skills on antibiotic use in lower respiratory tract infections: cluster randomised trial. BMJ 338, 1–10 (2009). 10.1136/bmj.b1374 PMC2677640 19416992

[bib3] Ishengoma, D. et al. Health laboratories in the Tanga region of Tanzania: the quality of diagnostic services for malaria and other communicable diseases. Ann. Trop. Med. Parasitol. 103, 441–453 (2009). 19583914 10.1179/136485909X451726

[bib4] Davies, J. & Davies, D. Origins and evolution of antibiotic resistance. Microbiol. Mol. Biol. Rev. 74, 417–433 (2010). 20805405 10.1128/MMBR.00016-10 PMC2937522

[bib5] Laxminarayan, R. et al. Antibiotic resistance-the need for global solutions. Lancet Infect. Dis. 13, 1057–1098 (2013). 24252483 10.1016/S1473-3099(13)70318-9

[bib6] Little, P. et al. Amoxicillin for acute lower-respiratory-tract infection in primary care when pneumonia is not suspected: a 12-country, randomised, placebo-controlled trial. Lancet Infect. Dis. 13, 123–129 (2013). 23265995 10.1016/S1473-3099(12)70300-6

[bib7] Butler C. et al. Variation in antibiotic prescribing and its impact on recovery in patients with acute cough in primary care: prospective study in 13 countries. BMJ 2009; 338: b2242. 19549995 10.1136/bmj.b2242 PMC3272656

[bib8] Goossens, H. , Ferech, M. , Vander-Stichele, R. & Elseviers, M. Outpatient antibiotic use in Europe and association with resistance: a cross-national database study. Lancet 365, 579–587 (2005). 15708101 10.1016/S0140-6736(05)17907-0

[bib9] Canton, R. et al. Rapid evolution and spread of carbapenemases among Enterobacteriaceae in Europe. Clin. Microbiol. Infect. 18, 413–413 (2012). 22507109 10.1111/j.1469-0691.2012.03821.x

[bib10] Welday, A. , G/Meskel, E. & Sileshi, M . Tigray Regional Health Bureau 2012 Annual Health Profile. Mekelle: The Government of Tigray National Regional State, Bureau of Health, (2012).

[bib11] Africa-Health-Workforce-Observatory. Africa Health Workforce Observatory. Health Resource for Health: Country Profile of Ethiopia. Addis Ababa, (2010).

[bib12] Beyene W. , Sudhakar M. Assessment of quality of health-care in jimma zone, southwest ethiopia. Ethiop. J. Health Sci. 21(Suppl): 49–58 (2011). 22435008 PMC3275883

[bib13] Cals, J. , Schot, M. , de-Jong, S. , Dinant, G. -J. & Hopstaken, R. Point-of-care c-reactive protein testing and antibiotic prescribing for respiratory tract infections: a randomized controlled trial. Ann. Fam. Med. 8, 124–133 (2010). 20212299 10.1370/afm.1090 PMC2834719

[bib14] Hopstaken, R. et al. Contributions of symptoms, signs, erythrocyte sedimentation rate and C-reactive protein to a diagnosis of pneumonia in acute lower respiratory tract infection. Br. J. Gen. Pract. 53, 358–364 (2003). 12830562 PMC1314594

[bib15] Van der Meer, V. N. A. , van den Broek, P. J. & Assendelft, W. J. Diagnostic value of C reactive protein in infections of the lower respiratory tract: systematic review. BMJ 331, 26–29 (2005). 15979984 10.1136/bmj.38483.478183.EB PMC558535

[bib16] Falk, G. & Fahey, T. C-reactive protein and community-acquired pneumonia in ambulatory care: systematic review of diagnostic accuracy studies. Fam. Pract. 26, 10–21 (2009). 19074757 10.1093/fampra/cmn095

[bib17] Little, P. et al. Effects of internet-based training on antibioitic prescribing rates for acute respiratory-tract infection: a multinational, cluster, randomised, factorial, controlled trial. Lancet 382, 175–182 (2013). 10.1016/S0140-6736(13)60994-0 PMC3807804 23915885

[bib18] Cals, J. et al. C-reactive protein point-of-care testing for lower respiratory tract infections: a qualitative evaluation of experiences by GPs. Fam. Pract. 27, 212–218 (2010). 20022909 10.1093/fampra/cmp088

[bib19] Nicks, B. , Manthey, D. & Fitch, M. The Centers for Medicare and Medicaid Services (CMS) community-acquired pneumonia coremeasures lead to unnecessary antibiotic administration by emergency physicians. Acad. Emerg. Med. 16, 184–187 (2009). 19133854 10.1111/j.1553-2712.2008.00320.x

[bib20] Zecca, E. et al. Reliability of two different bedside assays for C-reactive protein in newborn infants. Clin. Chem. Laboratory Med. 47, 1081–1084 (2009). 10.1515/CCLM.2009.246 19728849

[bib21] Verlee L. et al. [Summary of NHG practice guideline 'Acute cough']. Ned. Tijdschr. Geneeskd. 156: A4188 (2012). 22917039

[bib22] Woodhead, M. et al. Pneumonia: diagnosis and management of community- and hospital-acquired pneumonia in adult: National Institute for Health and Care Excellence. Available at https://www.nice.org.uk/guidance/cg191. Accessed March 2015 . 25520986

